# Phase Transformations and Phase Segregation during Potassiation of Sn*_x_*P*_y_* Anodes

**DOI:** 10.1021/acs.chemmater.2c01570

**Published:** 2022-08-01

**Authors:** Andrew W. Ells, Matthew L. Evans, Matthias F. Groh, Andrew J. Morris, Lauren E. Marbella

**Affiliations:** † Department of Chemical Engineering, Columbia University, 500 W 120th Street, New York, New York 10027, United States; ‡ Theory of Condensed Matter Group, Cavendish Laboratory, University of Cambridge, J. J. Thomson Avenue, Cambridge CB3 0HE, United Kingdom; § School of Metallurgy and Materials, University of Birmingham, Edgbaston, Birmingham B15 2TT, United Kingdom; ∥ Institute for Inorganic Chemistry, RWTH Aachen University, Aachen 52074, Germany; ⊥ Institut de la Matière Condensée et des Nanosciences, UCLouvain, Chemin des Étoiles 8, Louvain-la-Neuve 1348, Belgium

## Abstract

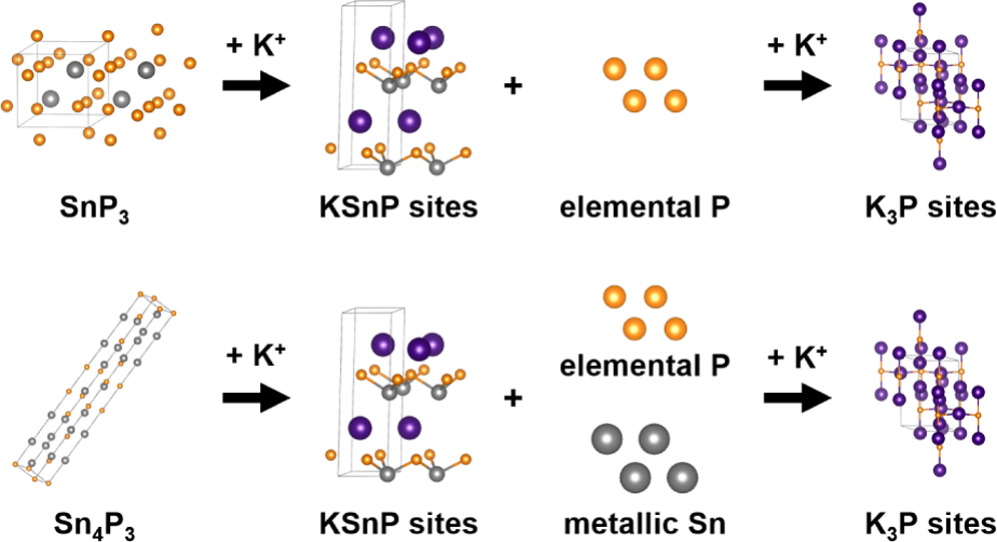

K-ion batteries (KIBs) have the potential to offer a cheaper alternative to Li-ion batteries (LIBs) using widely abundant materials. Conversion/alloying anodes have high theoretical capacities in KIBs, but it is believed that electrode damage from volume expansion and phase segregation by the accommodation of large K-ions leads to capacity loss during electrochemical cycling. To date, the exact phase transformations that occur during potassiation and depotassiation of conversion/alloying anodes are relatively unexplored. In this work, we synthesize two distinct compositions of tin phosphides, Sn_4_P_3_ and SnP_3_, and compare their conversion/alloying mechanisms with solid-state nuclear magnetic resonance (SSNMR) spectroscopy, powder X-ray diffraction (XRD), and density functional theory (DFT) calculations. *Ex situ*^31^P and ^119^Sn SSNMR analyses reveal that while both Sn_4_P_3_ and SnP_3_ exhibit phase separation of elemental P and the formation of KSnP-type environments (which are predicted to be stable based on DFT calculations) during potassiation, only Sn_4_P_3_ produces metallic Sn as a byproduct. In both anode materials, K reacts with elemental P to form K-rich compounds containing isolated P sites that resemble K_3_P but K does not alloy with Sn during potassiation of Sn_4_P_3_. During charge, K is only fully removed from the K_3_P-type structures, suggesting that the formation of ternary regions in the anode and phase separation contribute to capacity loss upon reaction of K with tin phosphides.

## Introduction

Conversion- and alloying-type anodes offer high capacities in alkali metal batteries, such as commercially successful Si anodes in Li-ion batteries (LIBs). Due to the greater abundance of K compared to Li (and the ability to use aluminum current collectors), K-ion batteries (KIBs) have the potential to lower the cost of grid-scale electrochemical storage^1−3^ and reduce demand pressure on Li. However, KIBs are still a nascent technology compared to LIBs, requiring new avenues of electrode and electrolyte materials design.^4−7^ K-ion conversion electrodes that can accommodate the large volume changes associated with K insertion/removal while simultaneously delivering practical energy densities are being actively developed. Phosphorus- and phosphide-based compounds display high theoretical specific capacity for KIBs,^8−12^ making these materials attractive to study as potential anodes. In particular, binary metal phosphides provide a route to mitigate the large volume expansion observed upon conversion reactions with phosphorus (up to 600% for the formation of K_3_P) by producing ternary intermediates and/or displacement and subsequent conversion reactions during potassiation of the parent phase.^13−18^

Of the binary compounds, tin phosphides are readily synthesized in a variety of different stoichiometries (e.g., Sn_4_P_3_, SnP, Sn_3_P_4_, and SnP_3_^19,200^) offering a rich phase space to study the mechanisms underpinning potassiation/depotassiation in high-capacity (>600 mAh g^–1^) anodes. Yet, many of the intermediates generated upon K reaction with tin phosphides are highly disordered and separate into multiple phases, making structural assignment challenging. For example, Guo and co-workers used *operando* X-ray diffraction (XRD) to examine the potassiation behavior in Sn_4_P_3_, proposing a mechanism by which the Sn_4_P_3_ phase-separates into metallic Sn and amorphous P upon discharge to ∼0.21 V vs K^+^/K, allowing the formation of K_3_P_11_.^20^ After further discharge to potentials below 0.17 V vs K^+^/K, K_3_P_11_ reacts with additional K to form K_3_P and metallic Sn alloys with K to form KSn. However, each of these assignments relied on a singular, low-intensity reflection in the XRD pattern (12.71° (for KSn), 12.14° (for K_3_P_11_), and 14.27° (for K_3_P)). Li and co-workers also report segregation of Sn_4_P_3_ and Sn, followed by conversion and alloying reactions that form K_3_P, KSn, and K_4_Sn_23_, although these assignments rely on minor, low-intensity XRD reflections or lattice spacing measurements from high-resolution transmission electron microscopy (HRTEM) of the crystalline components.^21^ In another study, Park and co-workers reported on the final discharge products formed at the end of potassiation of SnP_3_ using *ex situ* XRD, HRTEM, and X-ray photoelectron spectroscopy (XPS).^22^ The authors proposed a reaction pathway that included phase separation of Sn particles from bulk SnP_3_ and a conversion reaction between the remaining P and K to form K_3_P. The Sn followed a two-step alloying reaction to first form K_4_Sn_23_ and then KSn. Yet only one reflection could be observed for each intermediate in the diffraction data, indicating that heterogeneity and disorder in the anode complicate structural analyses.

Experimental and computational tools that can identify and parse amorphous, multicomponent species with high chemical resolution are required to understand and control complex potassiation behavior in binary anodes. In this study, we use a combination of ^31^P and ^119^Sn solid-state nuclear magnetic resonance (SSNMR) spectroscopy and density functional theory (DFT) calculations of stability and simulated NMR spectra for a large set of hypothetical structures to elucidate the amorphous and short-range crystalline structure in products that form during potassiation/depotassiation of SnP_3_ and Sn_4_P_3_. Assignments of crystalline structures are supported via *ex situ* XRD at different states of discharge/charge of KIBs assembled with both anode materials. Both experiment and theory suggest that SnP_3_ and Sn_4_P_3_ exhibit distinct potassiation mechanisms. K insertion into Sn_4_P_3_ and SnP_3_ generates local environments resembling K-Sn-P ternary phases that are predicted to be stable based on DFT calculations. Displacement of metallic Sn is observed in the case of Sn_4_P_3_, but no Sn phase separates upon potassiation of SnP_3_. Unambiguous assignment of metallic Sn during K insertion is achieved by monitoring the metallic Knight shift in ^119^Sn NMR (metals give rise to unique NMR shifts that are well resolved from diamagnetic compounds due to coupling between nuclei and conduction electrons^23^). When metallic Sn is present, little to no K–Sn alloying is observed.

## Experimental Section

### Materials and Methods

Potassium metal (chunks in mineral oil, 98% trace metals basis), potassium hexafluorophosphate (KPF_6_, > 99.5%), propylene carbonate (PC, anhydrous, > 99%), dimethyl carbonate (DMC, anhydrous, > 99%), hexanes (anhydrous, > 99%), Sn (> 99%), and sodium carboxymethyl cellulose (CMC) were purchased from Sigma-Aldrich. Red phosphorous powder (98.9%) was purchased from Alfa Aesar. Carbon Super P was purchased from MTI Corporation. Prior to use, KPF_6_ salt was dried *in vacuo* overnight at 100 °C before bringing into an Ar-filled glovebox (O_2_ < 0.1 ppm, H_2_O < 0.5 ppm). All other materials were used as received.

### Synthesis of SnP_3_ and Sn_4_P_3_

SnP_3_ and Sn_4_P_3_ were prepared by first mixing Sn and red P in 1:3 and 4:3 molar ratios, respectively, in a stainless-steel ball mill in an Ar-filled glovebox. Powder mixtures were ball-milled in a SPEX 8000M Mixer/Mill for 8 h (SnP_3_) and 1 h (Sn_4_P_3_). Product purity was confirmed with XRD and SSNMR.

### Electrode Fabrication

Electrode films were created by mixing an 8:1:1 mass ratio of Sn*_x_*P*_y_*:carbon Super P:CMC binder. First, the Sn*_x_*P*_y_* and carbon Super P were mixed in a stainless-steel ball mill under Ar for 30 min. In a mortar and pestle, water was added dropwise (∼10 drops per 100 mg of dry mixture) to CMC binder until a slurry was formed. The Sn*_x_*P*_y_* and carbon mixture was then added and mixed to make a uniform slurry. The slurry was cast onto a Cu current collector (6 μm thick, MTI) using a 150 μm doctor blade and dried at 100 °C under vacuum overnight. The dried film was punched into 12.7 mm diameter disks to use in cell assembly. Typical mass loadings of active material (Sn*_x_*P*_y_*) per anode were 5–15 mg cm^–2^. To assemble the opposing K electrodes, K metal was first treated by rinsing thoroughly in hexanes, then removing the external oxide layer with a razor blade. Small pieces of potassium were then placed in a bag coated with hexanes and rolled into thin sheets (∼0.25 mm thick) using a cylindrical weight. The K sheet was then removed from the bag and, after waiting for the hexanes to evaporate, stamped into 12.7 mm diameter disks. These electrodes were used for all electrochemical testing and NMR characterization.

### Electrochemical Cycling

2032-type coin cell casings were used to assemble K/SnP_3_ and K/Sn_4_P_3_ half-cells with 15 mm diameter glass microfiber separators (purchased from GE Life Sciences) soaked with 0.8 M KPF_6_ in PC. Galvanostatic cycling experiments were performed at C/200 and C/100 for SnP_3_ and Sn_4_P_3_, respectively (where *n*C refers to full theoretical discharge in 1/*n* h). C-rates were calculated from the theoretical capacities of SnP_3_ and Sn_4_P_3_ for the formation of K_3_P and KSn (1266 and 613 mAh g^–1^, respectively). Cells were discharged to 0.05 V and charged to 2.00 V vs K^+^/K.

### Electrode Extraction

Table 1 summarizes the states of charge at which galvanostatic cycling was stopped for electrode extraction and characterization. After cycling, the Sn*_x_*P*_y_* electrode was removed from the coin cell and submerged in DMC for 30 s before drying at room temperature under vacuum for 30 min. Extraction was performed in an Ar-filled glovebox within, at most, 4 h after cells completed cycling. To collect data for the pristine electrodes, Sn*_x_*P*_y_* electrodes were assembled into coin cells, then immediately removed, washed, and dried as described.

**Table 1:** States of Charge (V vs K^+^/K) Where Sn*_x_*P*_y_* Electrodes Were Extracted for Structural Analysis with XRD and NMR

		XRD	NMR
SnP_3_	discharge	1.50, 0.67, 0.53, 0.47, 0.35, 0.06	0.53, 0.06
	charge	1.77, 2.00	1.77, 2.00
Sn_4_P_3_	discharge	0.90, 0.26, 0.21, 0.18, 0.11, 0.06	0.18, 0.06
	charge	1.50, 2.00	1.50, 2.00

### X-ray Diffraction

XRD patterns were collected on a PANalytical XPERT3 powder diffractometer with Cu Kα radiation. To confirm synthesis, pristine SnP_3_ and Sn_4_P_3_ powders were removed from the Cu current collector and placed directly on a zero-background Si plate in the glovebox and sealed with Kapton polyimide film (Chemplex) in an air-free sample holder for data collection. To conduct *ex situ* measurements, individual Sn*_x_*P*_y_* electrodes (including the Cu current collector) were placed on a zero-background Si plate and sealed in the same manner. Reference powder diffraction patterns for SnP_3_ and Sn_4_P_3_ are from the Inorganic Crystal Structure Database (Collection Codes 16293 and 15014, respectively). Rietveld refinement was performed using the TOPAS Academic software package (version 7.12). The crystallite sizes were estimated by a Voigt-convolution approach according to Balzar et al., assuming a lognormal size distribution.^24,25^

### One-Dimensional (1D) NMR Measurements

SSNMR experiments were performed at room temperature on a Bruker Avance NEO 600 MHz spectrometer equipped with a 1.6 mm HFXY magic-angle spinning (MAS) Phoenix NMR probehead. Sn*_x_*P*_y_* electrodes at various states of charge were washed and dried as described above. The Sn*_x_*P*_y_* active material was then scraped from the Cu current collector, ground in a mortar and pestle, and packed in a 1.6 mm o.d. ZrO_2_ rotor in an Ar-filled glovebox. 1D ^31^P NMR (90° single pulse, 10 s recycle delay, 2048 scans) experiments were referenced to the ^31^P shift of solid ammonium dihydrogen phosphate at 0.8 ppm. The recycle delay for ^31^P NMR experiments was optimized on the pure phase binary compounds. ^119^Sn NMR was collected using a rotor-synchronized Hahn echo (90°−τ–180°−τ–acquire, where τ = 55.6 and 35.7 μs for 18 and 28 kHz MAS frequency, respectively) pulse sequence. ^119^Sn NMR was collected at two separate offset frequencies: one corresponding to metallic Sn at 6894 ppm with 46,080 scans and 0.5 s recycle delay, and a second corresponding to diamagnetic Sn compounds and alloys at −153 ppm with 512 scans and 60 s recycle delay, unless otherwise noted. ^119^Sn NMR shifts were externally referenced to SnO_2_ at −604.3 ppm.

### Density Functional Theory Calculations

The ternary K–Sn–P composition space has been recently explored using various crystal structure prediction techniques, namely, *ab initio* random structure searching (AIRSS), evolutionary algorithms, and structural prototyping,^26,27^ yielding a wealth of predicted stable and metastable phases that could form during electrochemical cycling. Gauge Including Projector Augmented Waves (GIPAW) calculations were performed with CASTEP (v20)^28,29^ to predict the ^31^P chemical shifts for these stable and low-lying metastable phases of K–Sn–P reported in previous work.

The GIPAW-NMR calculations employed the PBE functional,^30^ Vanderbilt ultrasoft pseudopotentials,^31^ and a plane-wave cutoff energy of 650 eV. The Brillouin zone was consistently sampled with Monkhorst–Pack grids of maximum spacing of 2π × 0.02 Å^–1^.^32^ All structures were relaxed until the forces on each ion fell below 0.01 eV/Å^–1^ prior to performing the chemical shielding calculations; any structures exhibiting metallicity at the PBE level of theory were excluded from the subsequent GIPAW calculations due to the difficulty of reliably simulating chemical shielding of paramagnetic systems.^33^ The computed chemical shielding tensors were referenced against reported experimental ^31^P shifts for black P, LiP_5_, Li_3_P, NaP, and Na_3_P to provide chemical shift tensors (Figure S9), showing good agreement (fit gradient of −0.997, *R*^2^, of 0.996).^34,35^ All of the described calculations and analyses were performed at high throughput with the open-source Python library matador.^26^

## Results

### Synthesis and Characterization of Tin Phosphide Anodes

**Figure 1:**
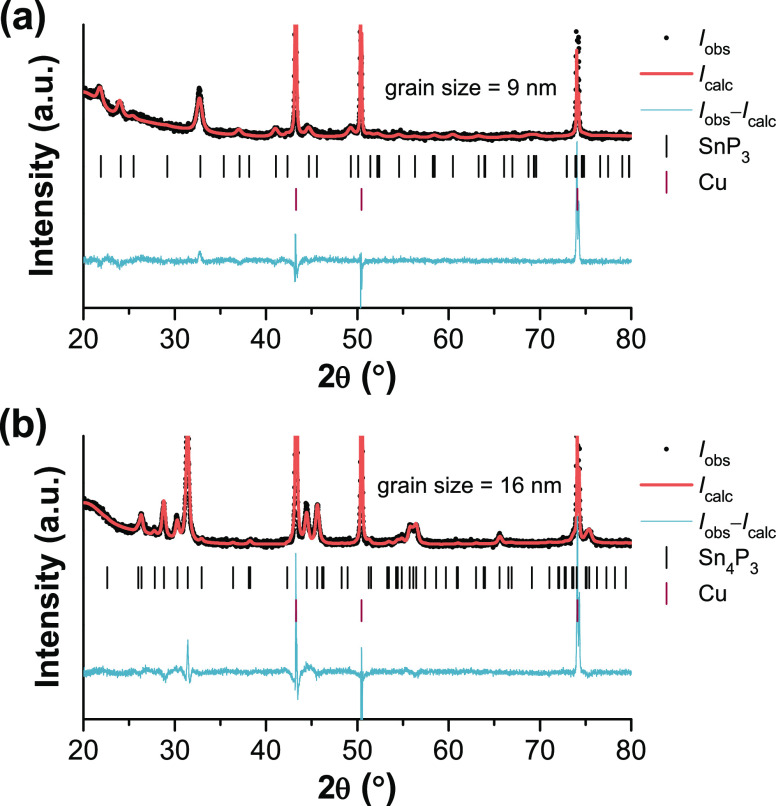
Rietveld refinement of the XRD pattern corresponding to (a) SnP_3_ and (b) Sn_4_P_3_ films after ball-milling with conductive carbon, mixing with CMC, and coating on Cu foil. The crystallite sizes were estimated using a Voigt-convolution approach according to ref (24), assuming a lognormal size distribution.

SnP_3_ and Sn_4_P_3_ were synthesized by ball-milling stoichiometric amounts of Sn and P. The structural integrity of SnP_3_ and Sn_4_P_3_ after ball-milling with conductive carbon for electrode fabrication was confirmed by XRD (Figure 1). Rietveld refinement of XRD patterns from the SnP_3_ and Sn_4_P_3_ films indicates that the average grain sizes of the active materials are 9 and 16 nm, respectively (assuming a lognormal distribution). Nano-sized active materials are consistent with previously reported syntheses for use in KIBs.^22,36^ Particles in this size range are expected to improve transport through the active material and may be less prone to fracture and pulverization during electrochemical cycling compared to micron-sized particles.^37^

### Electrochemical Characterization of Tin Phosphides and Possible Potassiation Pathways

**Figure 2:**
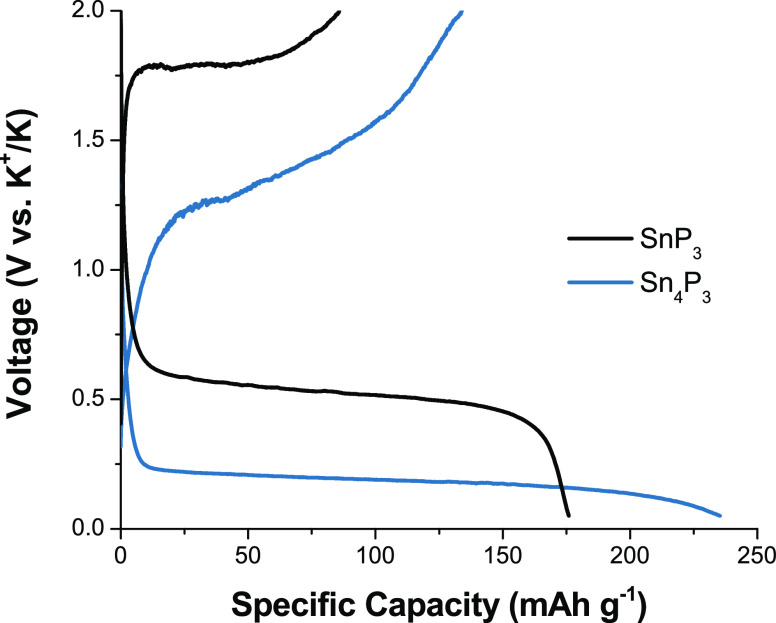
Voltage profiles of first-cycle discharge/charge for SnP_3_ (black line) and Sn_4_P_3_ (blue line) during galvanostatic cycling at C/200 and C/100, respectively.

The experimental galvanostatic discharge/charge plot (Figure 2) shows that tin phosphides exhibit first-cycle discharge capacities of 176 (for SnP_3_) and 235 mAh g^–1^ (for Sn_4_P_3_). The lower discharge capacity compared to the theoretical capacity is consistent with either deviation from the predicted potassiation pathway and/or sluggish kinetics that prevent K insertion in the active material. The shape of the voltage profile observed upon the charge of SnP_3_ is similar to the discharge curve, albeit at lower capacity, suggesting that potassiation may be only partially reversible and/or that side reactions occur on the first discharge. In contrast, the voltage profile for K removal from Sn_4_P_3_ is substantially more sloped, indicating that different phase transformations may occur during depotassiation. Voltage profiles for later cycles retain these features (Figure S1).

To understand the possible phase transformations taking place during potassiation/depotassiation of the tin phosphide anodes, we first turn to DFT. DFT calculations describe the thermodynamic stability of several phases in the relevant composition space, many of which are not currently reported in crystal structure databases, computational or otherwise (indicated with an asterisk (*) in the following lists). The full predicted phase space is elaborated elsewhere;^38^ in this work, the local environments from low-lying trial structures are used to provide insight into the short-range structural motifs formed during cycling and probed with SSNMR and XRD (vide infra).

No crystal structures for ternary K–Sn–P compounds exist in current databases. Our DFT calculations predict that KSn_2_P_2_, KSn_3_P_3_, KSnP, K_5_SnP_3_, and K_8_SnP_4_ are all stable phases that may be formed during potassiation or depotassiation of either Sn_4_P_3_ or SnP_3_ anodes (shown in Figure S2). For the K–P edge, each of KP_15_, KP_7_*, K_3_P_11_, K_3_P_7_*, K_2_P_3_, KP, and K_5_P_4_* are predicted to be stable. Most notably, calculations performed with the PBE functional also indicate that two possible charging endpoints, K_4_P_3_ and K_3_P, are both only metastable at finite temperatures; however, they both lie very close to the convex hull (+2 and +5 meV/atom, respectively). Applying instead the rSCAN functional,^39,40^ K_3_P is found to be thermodynamically stable, yet lies almost precisely on the existing tie-line between K_5_P_4_ and K, indicating that this phase may not form unless cycled to very low voltages (0.01 V vs K^+^/K). Similar calculations for the K–Sn phase space indicate that there are three stable K–Sn phases, namely, K_3_Sn_17_ (which destabilizes the known K_4_Sn_23_ phase), K_4_Sn_9_, and KSn.

We note that while DFT-predicted thermodynamic stability is useful for screening individual trial structures, the formation of a given phase during electrochemical cycling is highly dependent on kinetics.

### Experimental Characterization of Potassiation/Depotassiation Mechanisms of SnP_3_ and Sn_4_P_3_

**Figure 3:**
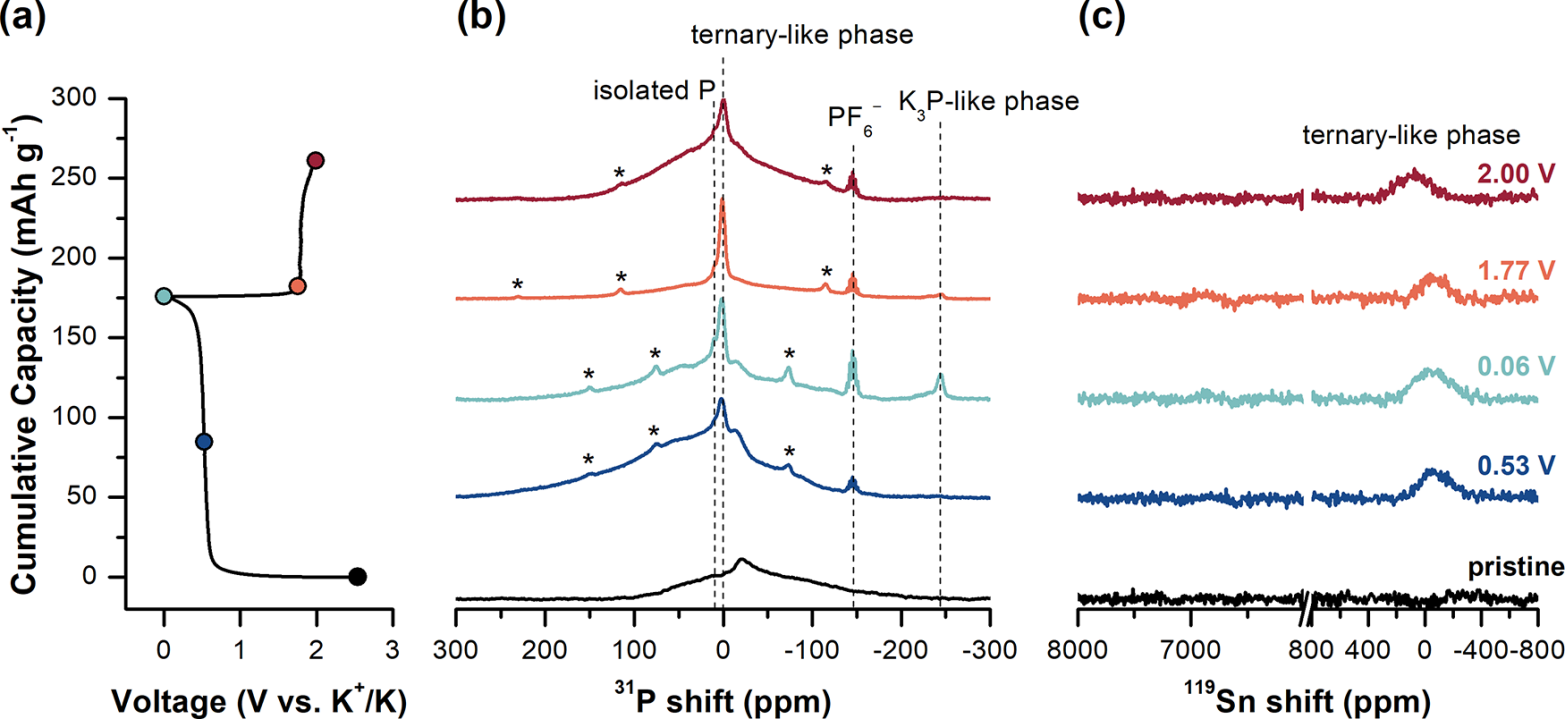
(a) Voltage profile of first discharge/charge of SnP_3_ under galvanostatic conditions at C/200. Colored points mark the states of charge at which cycling was stopped and SnP_3_ anodes were extracted for structural analysis. *Ex situ* (b) ^31^P and (c) ^119^Sn SSNMR of SnP_3_ anodes during the initial discharge (blue-hue spectra) and charge (red-hue spectra). The ^31^P quintet at −146 ppm (*J*_P–F_ = 675 Hz) is assigned to residual PF_6_^−^ from the electrolyte. Asterisks indicate spinning sidebands. Experiments were performed at either 18 or 28 kHz MAS frequency.

**Figure 4:**
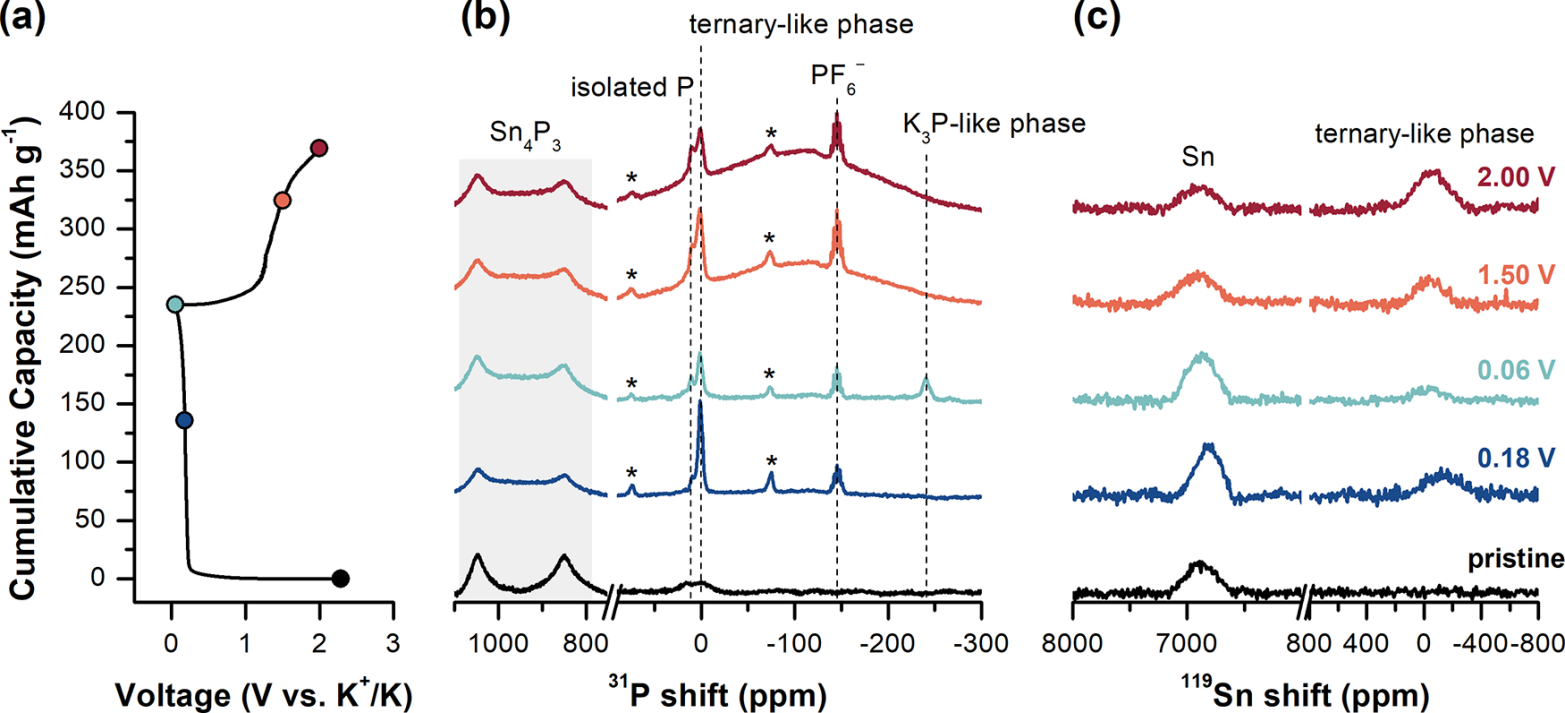
(a) Voltage profile of first discharge/charge of Sn_4_P_3_ under galvanostatic conditions at C/100. Colored points mark the states of charge at which cycling was stopped and Sn_4_P_3_ anodes were extracted for structural analysis. *Ex situ* (b) ^31^P and (c) ^119^Sn SSNMR of Sn_4_P_3_ anodes during the initial discharge (blue-hue spectra) and charge (red-hue spectra). The ^31^P quintet at −146 ppm (*J*_P–F_ = 675 Hz) is assigned to residual PF_6_^−^ from the electrolyte. All experiments were performed at 18 kHz MAS frequency.

*Ex situ*^31^P and ^119^Sn SSNMR were collected at multiple states of charge to directly measure the local structural environments present in tin phosphide anodes during potassiation/depotassiation (Figures 3 and 4). Pristine SnP_3_ (Figure 3, black) exhibits a broad ^31^P signal ranging from 100 to −210 ppm, in addition to a sharp (fwhm ∼12 ppm) resonance centered at −20 ppm. Since R3̅*m*-SnP_3_ only contains one independent P site, the combination of broad and sharp lines for pristine SnP_3_ likely arises from small particle sizes (∼9 nm) observed in XRD after prolonged ball milling during synthesis (8 h) and/or amorphous SnP_3_ compounds. For nano-sized materials, atoms in the particle core often display a sharp resonance consistent with the bulk crystalline material, whereas the surface sites exhibit inhomogeneous broadening.^41−43^ In the pristine state, ^119^Sn SSNMR does not show a resonance for SnP_3_ (Figure 3c, black), possibly due to long spin-lattice (*T*_1_) relaxation times, in combination with inhomogeneous line broadening, making detection prohibitively long^44^ (the maximum recycle delay tested in these experiments was 1000 s, and no signal was observed after a frequency sweep from 6400 to −9300 ppm).

After discharge of SnP_3_ to the center of the voltage plateau at 0.53 V vs K^+^/K (Figure 3, dark blue), a narrow ^31^P resonance at 2.6 ppm (fwhm ∼23 ppm) appears with a small shoulder at 11 ppm (these peaks are more clearly observed in Figure S6, which depicts the ^31^P spectra alone). The resonance at 11 ppm is consistent with elemental black P,^45^ whereas the peak at 2.6 ppm agrees with the ^31^P shift predicted for the on-hull **P**6_3_**mc**-KSnP ternary phase discovered with DFT (δ_iso_ = 2.3 ppm, Figure S10). Likewise, a broad ^119^Sn resonance at −66 ppm (fwhm ∼240 ppm) appears at the same voltage, supporting the formation of new Sn environments that may resemble KSnP-type sites. (N.B. Alkali metal insertion into Sn*_x_*P*_y_* likely decreases *T*_1_ relaxation times,^46−48^ allowing us to observe ^119^Sn resonances during potassiation). The lack of new Bragg reflections in XRD (Figure S3) indicates that crystalline KSnP does not form, but rather that K reaction with SnP_3_ results in local environments similar to those found in the ternary KSnP phase (where P is coordinated to three Sn and three K atoms and exhibits C_3v_ symmetry).

In the fully discharged state at 0.06 V vs K^+^/K (Figure 3, light blue), we continue to observe the ^31^P peak corresponding to black P, as well as a new peak at −244.5 ppm; no change is observed in ^119^Sn NMR. The ^31^P resonance at −244.5 ppm is similar to that observed for both Na_3_P (δ_iso_ = −207 ppm)^45^ and Li_3_P (δ_iso_ = −275 ppm).^49,50^ Examination of the chemical shift anisotropy (CSA) associated with the ^31^P resonance at −244.5 ppm shows that there is very little anisotropy at this site, consistent with the formation of isolated P atoms, likely surrounded by K, similar to those expected for K_3_P (Figure S7, experimental: Δ = 61 ppm, η = 0.07; calculated: Δ = 56 ppm, η = 0.42). These data indicate that phase separation of elemental P enables a conversion reaction between P and K that produces K_3_P-like structures. Since there are no additional reflections observed in XRD at the end of discharge (Figure S3), we suspect that these K_3_P-like environments do not exhibit long-range order. Further, we do not observe metallic Sn at any state of charge (Figure 3c; Figure S8 shows ^119^Sn NMR of bulk Sn), indicating that only reactions of K with P and Sn*_x_*P*_y_* lead to the observed capacity.

*Ex situ*^31^P and ^119^Sn NMR of the intermediates formed during potassiation of Sn_4_P_3_ are shown in Figure 4. The two resonances centered at 1050 and 850 ppm in the ^31^P SSNMR of the pristine sample (Figure 4b, black) are assigned to the two independent P sites (at the 6*c* and 3*a* positions, respectively) in R3̅*m*-Sn_4_P_3._^49^ Minor resonances in ^31^P and ^119^Sn NMR spectra are attributed to residual P (at ∼15 ppm) and Sn (center of mass at ∼6870 ppm) from synthesis. Upon discharge to the middle of the voltage plateau at 0.18 V vs K^+^/K (Figure 4, dark blue), ^31^P resonances at 2.6 and approximately 16 ppm are assigned to KSnP-like sites and phase-separated P, respectively. The presence of KSnP-like sites is again reflected in the ^119^Sn spectra by the resonance centered at −138 ppm; both ^31^P and ^119^Sn NMR spectroscopies are consistent with observations from SnP_3_ upon initial potassiation. Analysis of ^119^Sn NMR shows an increase in the NMR signal intensity for metallic Sn, indicating that Sn particles are produced during potassiation. The presence of metallic Sn is also confirmed with XRD, in which multiple small Sn reflections appear at 0.18 V (Figures S4 and S5). At full discharge to 0.06 V vs K^+^/K (Figure 4, light blue), K reacts with elemental P to form disordered K_3_P-type structures. During discharge/charge, the ^119^Sn NMR resonance for metallic Sn does not change in width, nor the center of mass, which strongly suggests that metallic Sn particles do not participate in further reactions. DFT and *ex situ* studies of ^119^Sn NMR during Li–Sn alloying reveal that isotropic ^119^Sn shifts are very sensitive to alloying (e.g., LiSn has two resonances at 5969 and 5429 ppm compared to a single resonance at 6915 ppm for pure β-Sn).^51,52^ We would expect similar ^119^Sn NMR spectrum changes if K–Sn alloying were taking place.

### Depotassiation Mechanisms of SnP_3_ and Sn_4_P_3_

Upon charge of SnP_3_ to 1.77 V vs K^+^/K (Figure 3, light red), the ^31^P resonance corresponding to the sites resembling K_3_P decreases considerably, while the relative ratio of elemental phosphorus (at 11 ppm) increases, consistent with K removal. At 2.00 V vs K^+^/K (Figure 3, dark red), the ^31^P NMR spectrum of fully charged SnP_3_ resembles that of the pristine material, as indicated by the shoulder at 40 ppm and the broad ^31^P NMR lineshape, but resonances corresponding to KSnP-type sites remain in both ^31^P and ^119^Sn NMR. The center of mass of the KSnP-type environment in the diamagnetic region of ^119^Sn NMR shifts to higher ppm (δ_2.00 V_ – δ_1.77 V_ = 150 ppm), likely due to changes in the stoichiometry of K*_x_*Sn*_y_*P*_z_*, some of which may regenerate SnP_3_-like sites. Partial reformation of SnP_3_ is also consistent with observations from XRD that show increased intensity for SnP_3_ reflections upon charging relative to the fully discharged electrode (Figure S3).

Upon depotassiation of Sn_4_P_3_ to 1.50 V vs K^+^/K (Figure 4, light red), K is readily removed from K_3_P-type environments, whereas KSnP-type sites persist, similar to SnP_3_. Again, K removal leads to small changes in the peak position of K*_x_*Sn*_y_*P*_z_* sites in ^119^Sn NMR and the emergence of a broad peak extending from 170 to −310 ppm in ^31^P NMR (center of mass = −110 ppm). DFT simulations predict several K*_x_*Sn*_y_*P*_z_* phases with ^31^P chemical shifts in this range, indicating that both Sn_4_P_3_ and SnP_3_ anodes generate highly disordered K*_x_*Sn*_y_*P*_z_* coordination environments, although these sites may overlap with inhomogeneous broadening observed for SnP_3_ particles. The chemical shift of metallic Sn in ^119^Sn NMR remains constant (Figure 4) during charge, providing evidence that phase-segregated Sn does not alloy with K.

## Discussion

The combination of SSNMR and DFT provides unique insight into the potassiation/depotassiation behavior of tin phosphide anodes. First, we assign a new ternary KSnP-type environment that forms upon discharge of the battery. The formation of KSnP-like coordination environments in both Sn_4_P_3_ and SnP_3_ is consistent with our predictions from DFT that indicate **P**6_3_**mc**-KSnP is thermodynamically stable upon potassiation of both parent compounds. However, differences in stoichiometry and structure between the two tin phosphides suggest that each has a distinct potassiation mechanism that forms these environments. In Sn_4_P_3_, the two-dimensional structure allows for topotactic intercalation and coordination of K with the alternating rows of Sn and P along the *c*-axis, forming Sn and P coordination environments similar to those found in the KSnP-like sites in NMR. This process leads to displacement of elemental P and concurrent reduction of Sn, but only P undergoes a conversion reaction upon subsequent potassiation. In contrast, the formation of KSnP-like sites in the three-dimensional SnP_3_ structure requires cleavage of P–P bonds during reduction. P–P bond cleavage during K insertion results in the phase separation of P observed in ^31^P NMR, ultimately producing local ^31^P sites that can coordinate to three nearby Sn atoms (bond distances of 2.72, 2.97, and 3.77 Å) and K to produce the C_3v_ symmetry about the P atom observed in KSnP. This mechanism resembles that proposed by Nazar and co-workers for Li insertion in MnP_4_, where reduction is facilitated by P–P bond breakage rather than redox chemistry associated with the metal center.^16^ In both tin phosphides, residual KSnP-like sites are present at full charge. This suggests kinetic or transport limitations on K removal and presents a mechanism of capacity fade by which K is locked in the tin phosphide structure. We note that this degradation is in addition to electrode cracking observed in scanning electron microscopy (SEM) of both tin phosphide anodes (Figure S11).

The second major finding of this work is that metallic Sn is only generated in the case of Sn_4_P_3_, and not in SnP_3_ as previously suggested.^22^ While this phase-separated Sn can, in principle, provide capacity in terms of K–Sn alloying (analogous to the behavior observed in tin phosphides for Li- and Na-ion batteries^49,53−57^), no evidence for the electrochemical reaction between K and Sn is seen in ^119^Sn NMR nor XRD. The lack of capacity from metallic Sn is consistent with a loss of electrical contact upon phase separation.^58^ Metallic Sn is never reincorporated in the anode, which is consistent with displacement reactions observed in LIB systems producing electrically isolated metal particles upon conversion and displacement reactions (e.g., InSb,^59^ Cu_2_Sb,^60^ CoP_3_^17^). Thus, most of the observed capacity in both Sn_4_P_3_ and SnP_3_ is due to the formation of disordered sites resembling K_3_P and likely explains the rapid capacity loss in these systems. Efforts to enable alloying anodes in KIBs must consider this phase separation and find a way to tether the components to the electrode during potassiation/depotassiation.

## Conclusions

The combination of SSNMR and DFT enabled the detection and assignment of short-range and amorphous intermediates previously invisible in diffraction analyses of tin phosphide anodes for KIBs. While the intention of binary phosphide anodes, such as tin phosphides, is to enable high capacity and mitigate volume expansion, we find that upon phase separation of elemental P (in both Sn_4_P_3_ and SnP_3_) and metallic Sn (in the case of Sn_4_P_3_), only P-containing compounds react reversibly with K. Local structures resembling KSnP persist after charge, suggesting transport and/or kinetic limitations for removing K from within the layered tin phosphides (regardless of starting stoichiometry). In the case of Sn_4_P_3_, the formation of electrically isolated Sn prevents total recovery of the pristine material and does not appear to add reversible capacity via alloying. The irreversible phase separation and structural rearrangements inherent to tin phosphides motivate new approaches to enabling P or binary phosphides as a high-capacity KIB anode material. We suspect that tin phosphides in KIBs may benefit from the approaches taken for Si–C anodes in LIBs,^61−63^ where nanoparticles of the high-capacity anode material (for KIBs, P) are embedded in a graphitic matrix to accommodate volume expansion.

## Supporting Information Available

Additional electrochemistry, structural models, XRD, solid-state NMR spectroscopy, simulated data, and scanning electron microscopy (Figures S1–S11) (PDF)

## Notes

NMR data may be found on the Open Science Framework at https://osf.io/xmcs5/. Computational data for K–Sn–P phase prediction may be found at https://doi.org/10.5281/zenodo.6546649.
