# Supplementary material for: Phase Transformations and Phase Segregation during Potassiation of SnxPy Anodes
Source: Chem Mater. 2022 Aug 1;34(16):7460–7. doi: 10.1021/acs.chemmater.2c01570 (PMC9404545; doi:10.1021/acs.chemmater.2c01570)
Supplement: Supplementary file 1 — cm2c01570_si_001.pdf [file cm2c01570_si_001.pdf]

Supporting Information

Phase transformations and phase segregation during potassiation of Sn_xP_y anodes

Andrew W. Ells,[†] Matthew L. Evans,[‡,¶] Matthias F. Groh[‖], Andrew J. Morris,[§*] and Lauren E. Marbella[†*]

†Department of Chemical Engineering, Columbia University, 500 W 120th St, New York, NY 10027

‡Theory of Condensed Matter Group, Cavendish Laboratory, University of Cambridge, J. J. Thomson Avenue, Cambridge CB3 0HE

§School of Metallurgy and Materials, University of Birmingham, Edgbaston, Birmingham B15 2TT, United Kingdom

‖Institute for Inorganic Chemistry, RWTH Aachen University, Aachen, Germany

¶ Institut de la Matière Condensée et des Nanosciences, UCLouvain, Chemin des Étoiles 8, Louvain-la-Neuve 1348, Belgium

Table of Contents

Electrochemistry……S2
Structure models……S3
X-ray diffraction……S4
Solid-state NMR spectroscopy……S7
Simulated data……S10
Scanning electron microscopy……S12

Electrochemistry

Despite cycling at low rates, both tin phosphide stoichiometries demonstrate poor cycling stability. SnP_3 retains 6.7% of its initial capacity after 10 cycles, and Sn_4P_3 retains 3.2%.

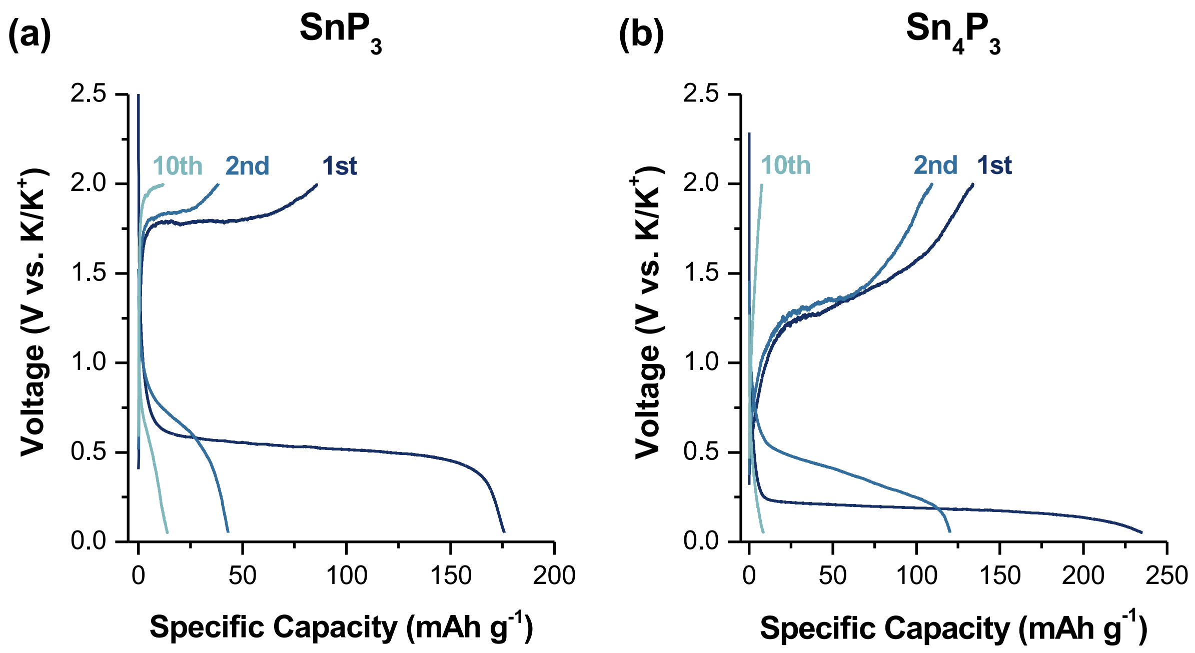

Figure S1. Voltage profiles of SnP_3 (a) and Sn_4P_3 (b) during the 1st, 2nd, and 10th cycles. Cells were galvanostatically cycled at C/200 and C/100, respectively.

Structure models

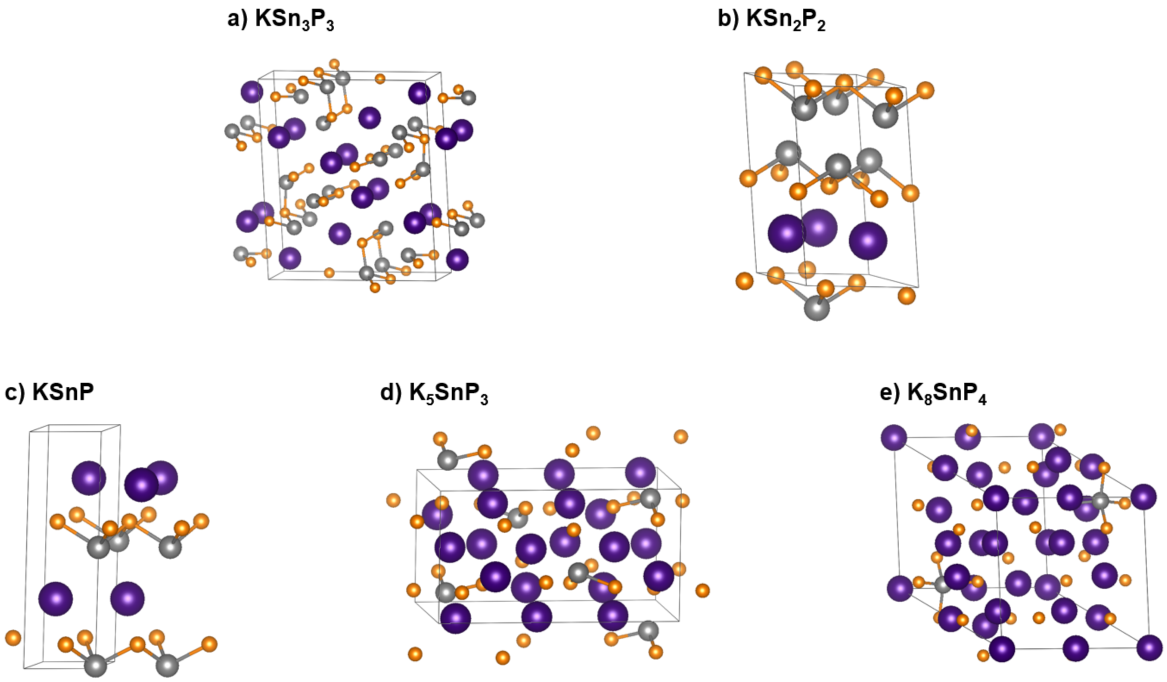

Figure S2. Predicted structures for ternary K-Sn-P structures found to be stable. K is shown in purple, P in orange, and Sn in gray.

X-ray diffraction

XRD patterns were collected at multiple states of discharge/charge for SnP_3 and Sn_4P_3 (Figures S3 and S4) in an attempt to identify products of potassiation/depotassiation. Pristine SnP_3 electrodes exhibit reflections that are consistent with the calculated pattern for rhombohedral SnP_3 ($R\bar{3}m$) (Figure S3). The reflections that correspond to SnP_3 decrease in intensity until the end of discharge at 0.06 V vs. K^+/K, with no measurable change in 2θ nor new Bragg reflections. The lack of new reflections during discharge suggests that the products of potassiation do not have long-range crystalline structures. During charge, the reflections corresponding to SnP_3 increase again, indicating that some SnP_3 reforms during depotassiation.

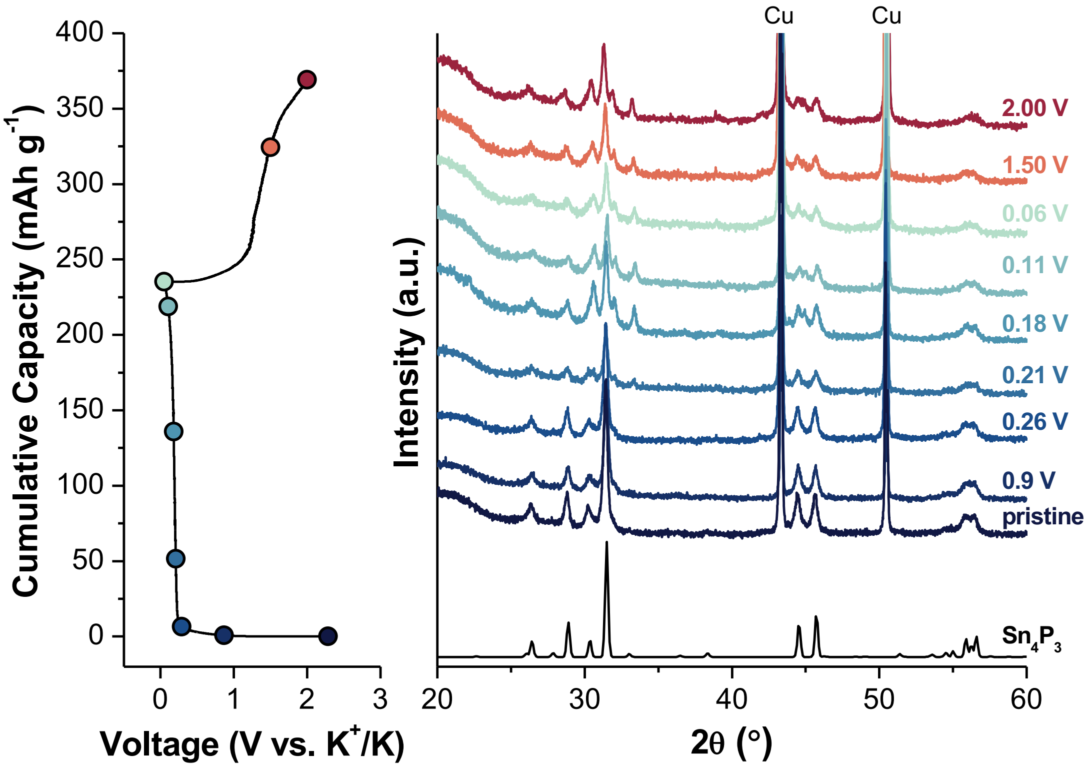

Figure S3. *Ex situ* XRD patterns of SnP_3 collected at different stages of the first potassiation/depotassiation cycle and the corresponding electrochemistry. The multicolored circles on the voltage profiles indicate where galvanostatic cycling was stopped and electrodes were removed from K half cells for XRD measurement. The XRD patterns from ICSD for the as-synthesized anodes is shown in black at the bottom. The broad signal from 20° to 23° corresponds to the polyimide film used to seal the air-sensitive samples.

Figure S4 shows that the XRD pattern for the pristine Sn_4P_3 electrode is consistent with the calculated pattern for rhombohedral Sn_4P_3 ($R\bar{3}m$). During discharge, Sn_4P_3 reflections decrease in intensity as the active material is partially, but not completely, consumed. During charge, reflections corresponding to pristine Sn_4P_3 reappear, suggesting the pristine material is at least partially regenerated. Figure S7 shows the patterns observed from 25° to 50° for select states of charge. Reflections at 30.6°, 32.0°, 43.9°, and 44.9° corresponding to metallic Sn first appear at 0.18 V and persist on charge without significant changes in Bragg angle. Rietveld refinement suggests an approximately 33:1 ratio between remaining Sn_4P_3 and crystalline Sn at 0.06 V. An additional new reflection at 33.4° cannot be indexed to any known K–P binary phase, K–Sn alloy, or tertiary K–Sn–P phase.

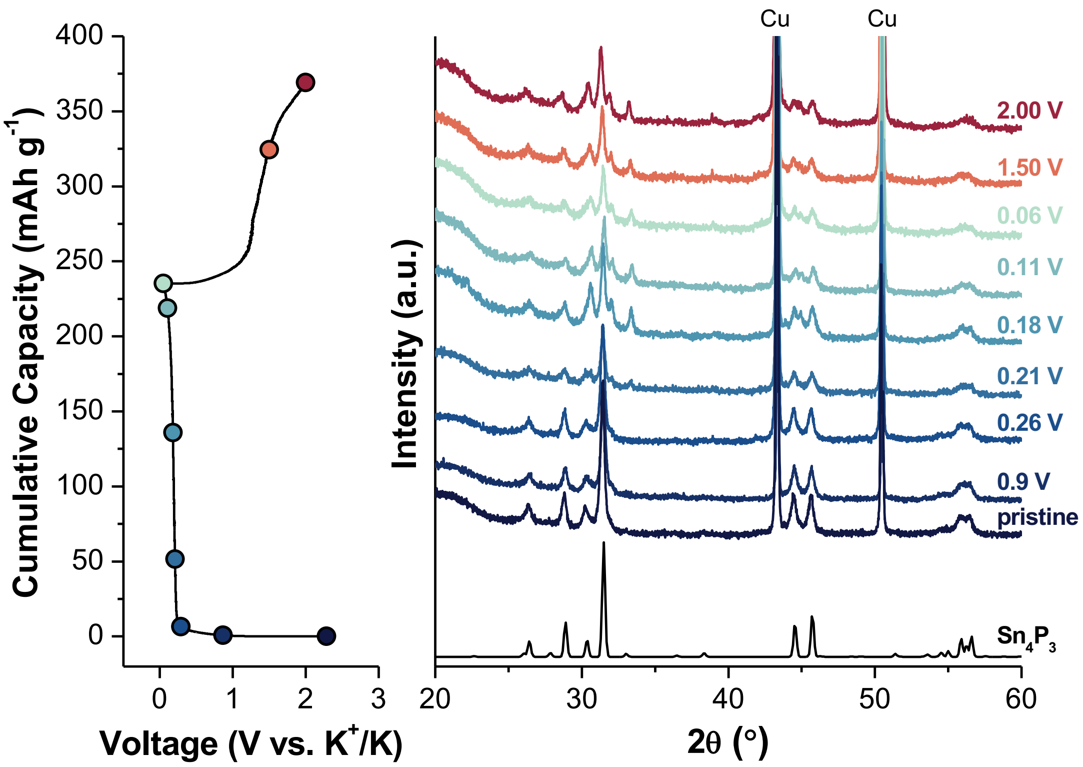

Figure S4. *Ex situ* XRD patterns of Sn_4P_3 collected at different stages of the first potassiation/depotassiation cycle and the corresponding electrochemistry. The multicolored circles on the voltage profiles indicate where galvanostatic cycling was stopped and electrodes were removed from K half cells for XRD measurement. The XRD patterns from ICSD for the as-synthesized anodes is shown in black at the bottom. The broad signal from 20° to 23° corresponds to the polyimide film used to seal the air-sensitive samples.

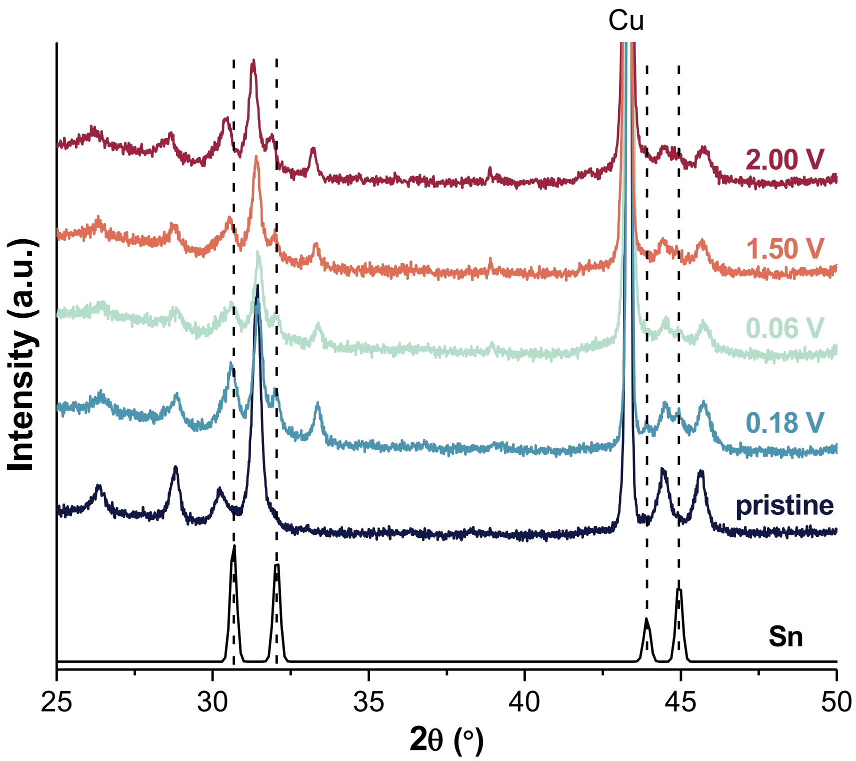

Figure S5. *Ex situ* XRD patterns of Sn_4P_3 collected at different stages of the first potassiation/depotassiation cycle, as well as metallic Sn (Inorganic Crystal Structure Database Collection Code 106072).

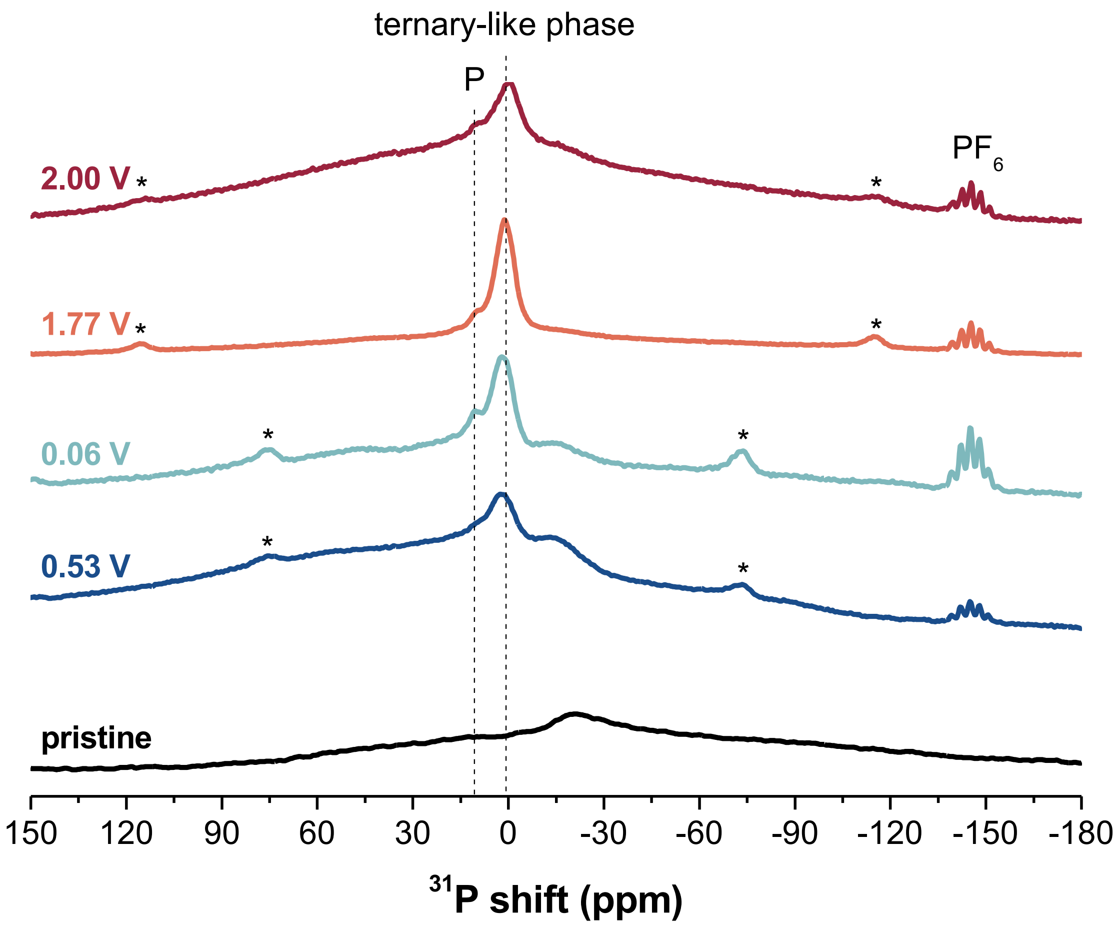

Figure S6. *Ex situ* ^{31}P SSNMR of SnP_3 anodes during the initial discharge (blue-hue spectra) and charge (red-hue spectra). The ^{31}P quintet at −146 ppm (J_{P-F} = 675 Hz) is assigned to residual PF_6 from the electrolyte. Asterisks indicate spinning sidebands. Experiments were performed at either 18 or 28 kHz MAS frequency.

2D phase-adjusted spinning sidebands (PASS) NMR experiments were performed at room temperature at a MAS frequency of 8 kHz. The PASS pulse sequence consists of a π/2 pulse followed by a train of five rotor-synchronized π pulses with interpulse delays that satisfy the PASS equations.[1] For each experiment, the indirect dimension was incremented in 32 steps, with 204 scans collected per step. All 2D data sets were processed by repeating the 2D signal in the indirect dimension eight times to separate spinning sidebands. All spinning sideband patterns were simulated in dmfit[2] to extract the principal components (δ_{11}, δ_{22}, and δ_{33}) of the chemical shift tensor for comparison to DFT models. All chemical shift tensors reported here use the Haeberlen–Mehring convention to define the isotropic chemical shift (δ_{iso}), anisotropy (Δ), and asymmetry (η) as follows:

$$\delta_{iso} = \left(\frac{\delta_{11} + \delta_{22} + \delta_{33}}{3}\right) \tag{1}$$

$$\Delta = \delta_{zz} - \left(\frac{\delta_{xx} - \delta_{zz}}{2}\right) = 3\left(\frac{\delta_{xx} - \delta_{iso}}{2}\right) \tag{2}$$

$$\eta = \left(\frac{\delta_{yy} - \delta_{xx}}{\delta_{zz} - \delta_{iso}}\right) \tag{3}$$

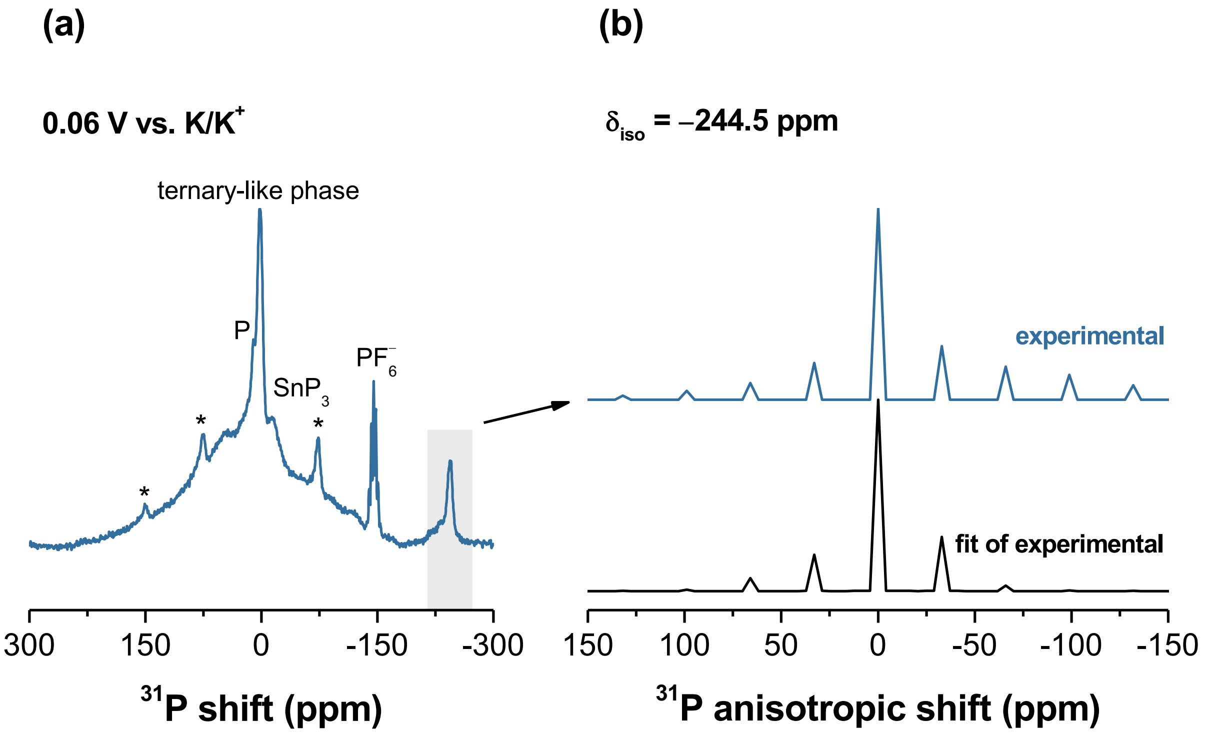

Figure S7. (a) ^{31}P SSNMR of SnP_3 fully discharged to 0.06 V. Asterisks indicate spinning sidebands. (b) Anisotropic projection of δ_{iso} = −244.5 ppm collected from 2D ^{31}P PASS NMR (blue) and simulated fit of experimental data (black).

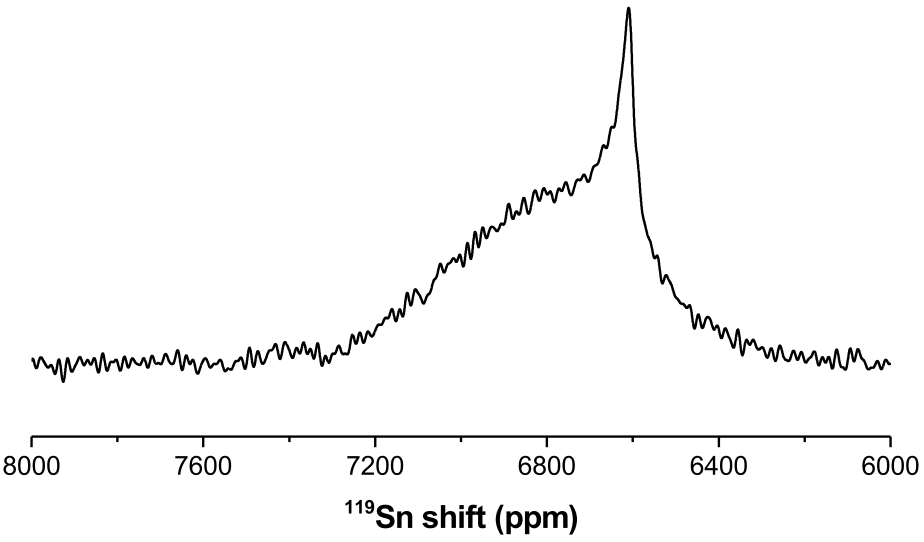

Figure S8. Static ^{119}Sn SSNMR of bulk metallic Sn.

Simulated data

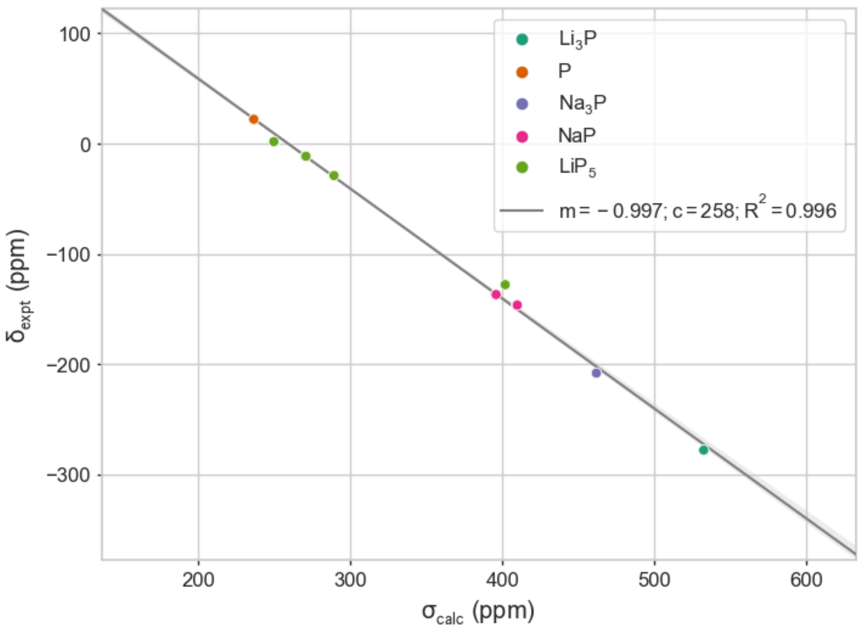

Figure S9. Referencing of ^{31}P chemical shielding (σ_{calc}) computed with CASTEP v20 under the GIPAW-NMR formalism. Measured chemical shifts (δ_{expt}) were taken from the literature for crystallographic sites in phases spanning the relevant chemical shift range (black P, Li_3P, Na_3P, NaP and LiP_5) and a least squares fit (weighted by the number of unique environments in each phase) was performed between the computed shieldings and measured shifts to provide a linear model (m=-0.997, c=258, R^2=0.996) to apply to the computed chemical shielding values of the hypothetical phases. The best fit line is shown in dark grey, with confidence intervals in light grey.

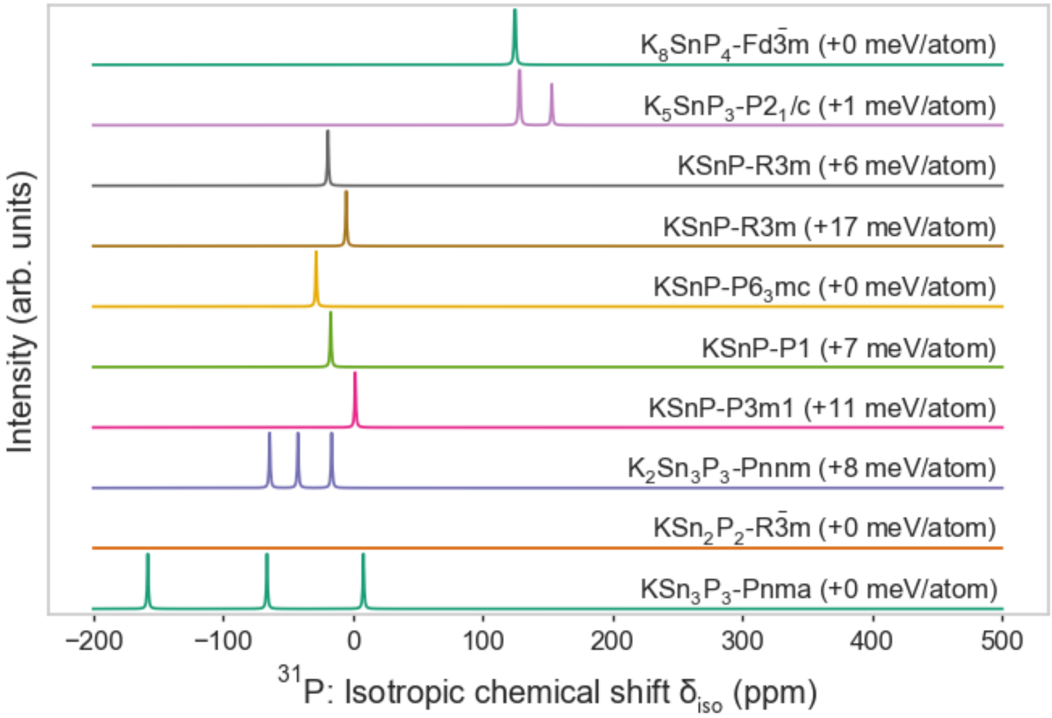

Figure S10. Computed isotropic chemical shifts (δ_{iso}) for hypothetical ternary K-Sn-P phases, labelled by their chemical formula, spacegroup and predicted distance from the convex hull at 0 K using the PBE functional.

Scanning electron microscopy

SEM was conducted using a Zeiss Sigma VP Schottky Thermal Field emission SEM with a Gemini objective lens. Cycled electrodes were prepared by triple rinsing in DMC to prevent charging under the electron beam. Samples were then dried under vacuum overnight to remove residual electrolytes and solvents. Pristine electrodes were not rinsed. Samples were mounted on SEM stubs inside of the glovebox using C tape. Samples were transferred to the SEM sample chamber using an airtight Ar-filled jar, with exposure to the atmosphere estimated to be < 10 s for each sample.

In SnP_3, electrode cracking is clearly present in active material particles after the first cycle (center of Figure S11b). In Sn_4P_3, individual active material particles are difficult to distinguish, but cracking of the electrode film after cycling is evident toward the left side of Figure S11d.

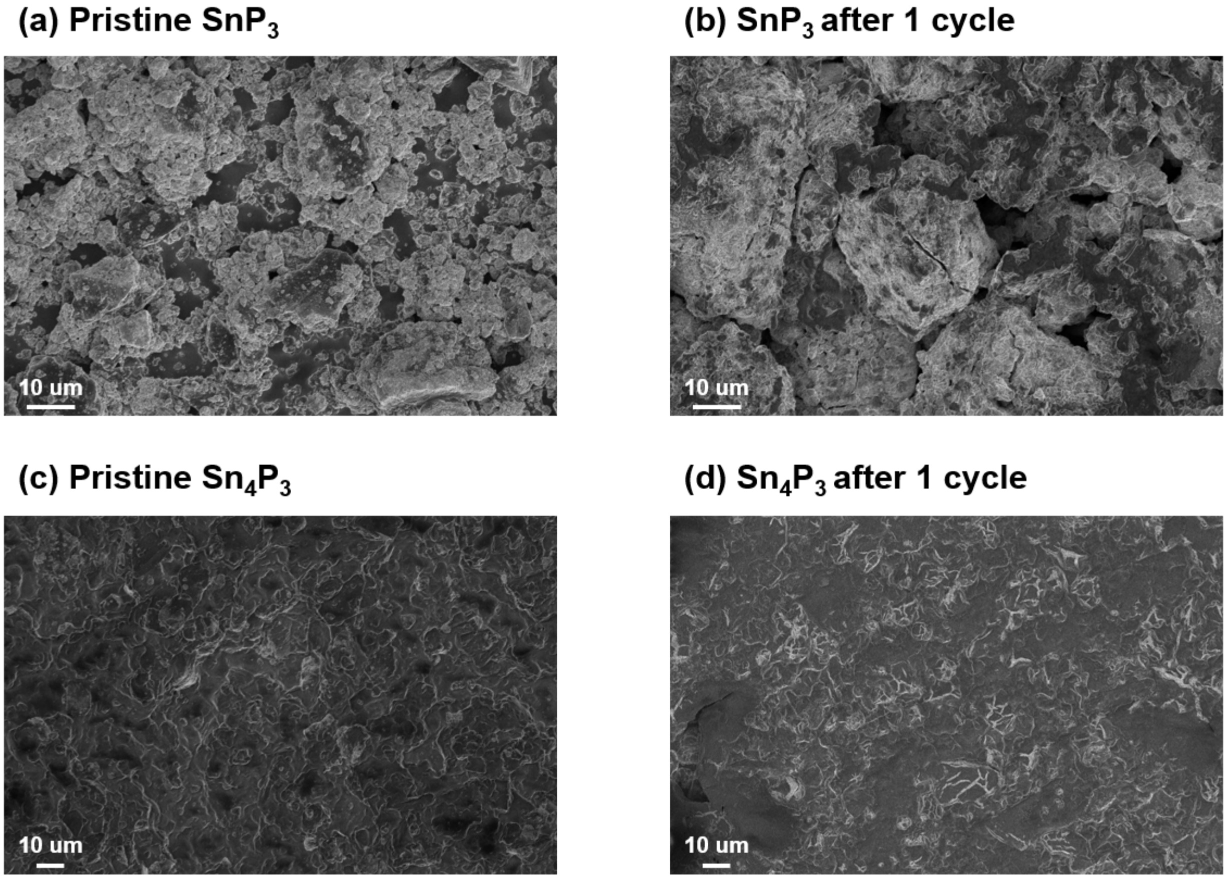

Figure S11. SEM images taken of a SnP_3 electrode before (a) and after (b) cycling, compared to a Sn_4P_3 electrode before (c) and after (d) cycling.

References

(1) Antzutkin, O. N.; Shekar, S. C.; Levitt, M. H. Two-Dimensional Sideband Separation in Magic-Angle-Spinning NMR. *Journal of Magnetic Resonance, Series A*. **1995**, 7–19.

(2) Massiot, D.; Fayon, F.; Capron, M.; King, I.; Le Calvé, S.; Alonso, B.; Durand, J. O.; Bujoli, B.; Gan, Z.; Hoatson, G. Modelling One- and Two-Dimensional Solid-State NMR Spectra. *Magn. Reson. Chem.* **2002**, *40*, 70–76.
